# The genetic diversity and relationships of cauliflower (*Brassica oleracea* var. *botrytis*) inbred lines assessed by using SSR markers

**DOI:** 10.1371/journal.pone.0208551

**Published:** 2018-12-06

**Authors:** Shiyang Zhu, Xiaoling Zhang, Qing Liu, Tiankuan Luo, Zheng Tang, Yuanchang Zhou

**Affiliations:** 1 Key Laboratory of Ministry of Education for Genetics, Breeding and Multiple Utilization of Crops, College of Crop Science, Fujian Agriculture and Forestry University, Fuzhou, P.R. China; 2 Key Laboratory of Crop Breeding in South Zhejiang, Wenzhou Vocational College of Science and Technology, Wenzhou, P.R. China; United States Department of Agriculture, UNITED STATES

## Abstract

Inbred lines are important germplasm in cauliflower breeding programs. To understand the genetic diversity and relationships of cauliflower inbred lines, the use of simple sequence repeat (SSR) markers will be of great value for parental line selection and breeding strategy design. In this study, the genetic diversity and relationships of 165 cauliflower inbred lines primarily derived from southeast China were assessed using SSR markers. Forty-three SSR markers were polymorphic across these inbred lines and generated a total of 111 alleles. The mean values of the number of alleles (Na), effective number of alleles (Ne), Shannon’s Information index (I), and polymorphism information content (PIC) per locus were 2.581, 1.599, 0.517 and 0.316, respectively. Genetic distance values among all pairs of the inbred lines varied from 0 to 0.67 with an average of 0.30. On the basis of genetic distance data estimated with the SSR markers, the 165 cauliflower inbred lines were classified into four main clusters (from group Ⅰ to group Ⅳ) by cluster analysis and four subpopulations (from POP 1 to POP 4) by structure analysis. The classification patterns of most cauliflower inbred lines were not consistent with their curd maturity, curd solidity or geographic origins. These results based on estimates by the SSR markers, suggested the genetic diversity of the 165 cauliflower inbred lines was relatively narrow. Therefore, pyramiding the valuable genes among different types of the cauliflower inbred lines is important to increase the genetic diversity to obtain desirable hybridization combinations. The information generated in this report will be useful for assessing germplasm and breeding in cauliflower.

## Introduction

Cauliflower (*Brassica oleracea* var. *botrytis*) is one of the important horticultural crops with an annual global production of over 25 million tons [1]. As an economical and nutritional vegetable crop, cauliflower is widely cultivated in India, China, Ecuador, America, Spain, and Mexico, among others. Thus, cauliflower has received much attention by breeders to improve agronomic characters, and some elite inbred lines have been used in cauliflower breeding programs. Inbred lines are important breeding materials in cauliflower breeding programs. An understanding of the genetic diversity and relationships of cauliflower inbred lines will be useful for assessing germplasm and breeding in cauliflower [2–3].

Traditionally, the genetic diversity and relationships of germplasm have usually been assessed based on morphological data, pedigree data, or geographic information on the origin of the materials [4–6]. Currently, some studies on genetic diversity and relationships in cauliflower have been conducted with morphologic traits [7–14]. However, this method is affected by environmental variation and is time-consuming. Molecular markers can overcome these limitations and serve as powerful and reliable tools for assessing genetic diversity and relationships [15]. Therefore, recently, various molecular markers, such as RAPD, RFLP, ISSR, AFLP, SRAP and SSR have been used to examine the genetic diversity and relationships in cauliflower [15–22]. Among these various molecular markers, SSR markers are widely known for their high level of reproducibility and abundant multiallelic forms [15, 23]. Previously, some researchers analyzed the genetic relationships among cultivars of three botanical varieties of *B*. *oleracea* (cauliflower, broccoli and cabbage) using a limited number of SSR markers and found that cauliflower cultivars had low genetic diversity among the three botanical varieties of *B*. *oleracea* [2–3]. Additionally, different germplasms might also have different genetic backgrounds resulting in differences in genetic diversity and relationships.

Although some genetic diversity and relationship studies were conducted with SSR markers in cauliflower, few previous studies involved cauliflower inbred lines from China. We obtained many inbred lines by self-selecting during cauliflower breeding programs since 1997. However, these inbred lines are rarely effectively utilized in our breeding programs. To increase utilization of the cauliflower inbred lines, we assessed phenotypic variation and diversity of 165 inbred lines and found that these inbred lines exhibited a wide degree of variability for most phenotypic traits [14]. In this paper, we assessed the genetic diversity and relationships of the 165 cauliflower inbred lines using SSR markers, to obtain useful information on these inbred lines as parent materials for further utilization in future breeding programs.

## Materials and methods

### Plant material and DNA extraction

One hundred and sixty-five (165) cauliflower inbred lines were used for genetic diversity and relationship determination, which were bred by artificial self-selecting from different cauliflower cultivars collected from different markets (such as different seed stores, seed companies, or seed trade fairs). These cauliflower cultivars were primarily derived from southeast China and other regions: Fujian (66), Zhejiang (44), Taiwan (25), Shanghai (5), Hong Kong (4), Jiangxi and Japan (3 each), Henan and Hunan (2 each), Chongqing, Nederland and Italia (1 each), unknown region (8). The cauliflower inbred lines are stored in the Wenzhou Academy of Agricultural Sciences, Wenzhou, Zhejiang Province, China. Detailed information on the original accessions and the inbred lines is listed in S1 Table.

Total genomic DNA was extracted from young leaves of 3-week-old seedlings of each inbred line using the protocol of Shi and Hong with modification [24]. A leaf sample was crushed using a mortar and pestle with liquid N_2,_ and then added to a 1.5 ml centrifuge tube. Extraction buffer, 600 μl (100 mM Tris-HCl, pH8.0, 50 mM EDTA, pH8.0, 0.5 M NaCl, 1.5%SDS), was then added, and the tube was vortexed and incubated for 30 min in a 65°C water bath. The tube was cooled to room temperature, 600μl of chloroform-isoamyl alcohol (24:1) was added, and then tube was mixed gently for 30 min and centrifuged at 8,000 rpm for 15 min. The supernatant was transferred to another 1.5 ml tube, 700 μl of precold alcohol (-20°C) was added and the tube was mixed gently to precipitate the DNA. The mixture was centrifuged at 12,000 rpm for 6 min, and the supernatant was removed carefully. The DNA pellet was washed with 400μl of 70% (v/v) alcohol five times and then air-dried and resuspended in 200μl of ddH_2_O. The DNA concentration was adjusted to 50 ng/μl based on spectrophotometer readings (UV-2802PC; Unico (Shanghai) Instrument Co., LTD., Shanghai, China), and the DNA was preserved at -20°C.

### SSR primer selection

Brassica SSR primer pairs were obtained from the previous papers of Tonguç and Griffiths [2], Louarn et al. [21], Batley et al. [25], Gao et al. [26], Li [27], Ciancaleoni et al. [28] and public sources at *http*:*//www*.*brassica*.*info/resource/markers*.*php*. A total of 509 SSR primer pairs were prescreened for polymorphism with ten distinct commercial varieties of cauliflower (i.e., Limin60, Shuaixue80, Xinhua80, Xiamei60, Baiyu60, Xiuyu80, Xueguanzao55, Xiabao, Xueguan60, and Mingxue). Primers were excluded from the study when they did not produce different or unambiguous band sizes. A final set of 43 SSR primers were selected for further analysis (S2 Table).

### PCR amplification

The PCR reactions were conducted in a total volume of 10μl, which contained 20 ng of DNA template, 1×PCR, 0.2μM forward and reverse primers, 0.2 mM dNTPs, and 0.5 units of Taq DNA polymerase. The PCR amplifications were performed in a DNA Thermal Cylcer (Bio-Rad Laboratories, Inc., USA) as follows: an initial denaturation step for 5 min at 94°C; 40s at 94°C, 40s at annealing temperature relying on the primers, and 1 min at 72°C for 35 cycles, followed by an extension step of 10 min at 72°C. Then, the reaction was stopped at 6°C.

### Electrophoresis and fragment detection

Ten microliters of the final PCR amplification product was mixed with 1μl of loading buffer (40% sucrose, 0.25% bromophenol blue and 0.25% Xylene cyanol FF). Two microliters of the sample was loaded onto a 6% acrylamide-bisacrylamide gel (19:1) in 1×TBE, and electrophoresed at 150 volts for 100–150 min. The fragments were detected using a silver staining procedure as follows. The peeled gel was fixed with 10% ethanol containing 0.5% acetic acid solution for 12 min. The fixed gel was placed in 0.1% AgNO_3_ staining solution for 12 min. Then, the stained gel was wash twice with ddH_2_O and placed in 1.5% NaOH containing 1% formaldehyde solution for approximately 10 min to obtain the visible fragments.

### Data analyses

The DNA fragment size amplified by SSR primers was determined based on DNA molecular marker size (range 100bp to 2000bp). The number of alleles (Na), effective number of alleles (Ne), and Shannon’s Information index (I) per locus were estimated using POPGEN version 1.32 software [29]. The polymorphism information content (PIC) was estimated as follows: PIC = 1-Ʃ (*pi*)^2^, where *pi* is the frequency of *i*th allele in a population [2]. Nei’s (1972) genetic distance matrix calculated with NTSYS 2.10 software [30] was used to construct a dendrogram based on a neighbor-joining (NJ) method in MEGA 4.0 software [31]. Principal coordinate analysis (PCA) was conducted with the genetic distance matrix data using DCENTER and EIGEN procedures in NTSYS 2.10 software [30]. Population structure analysis among cauliflower inbred lines was determined using STRUCTURE version 2.2 [32–33]. Ten independent runs were performed for each K (testing from 2 to 10) using a burn-in of 50,000 iterations and followed by 100,000 iterations. The optimum number of populations (K value) was determined by the mean log-likelihood value over 10 runs at each K. When the mean log-likelihood value reached the maximum value in the model parameter K value, then a suitable K value was determined.

## Results

### Molecular variance analysis

**Fig 1:**
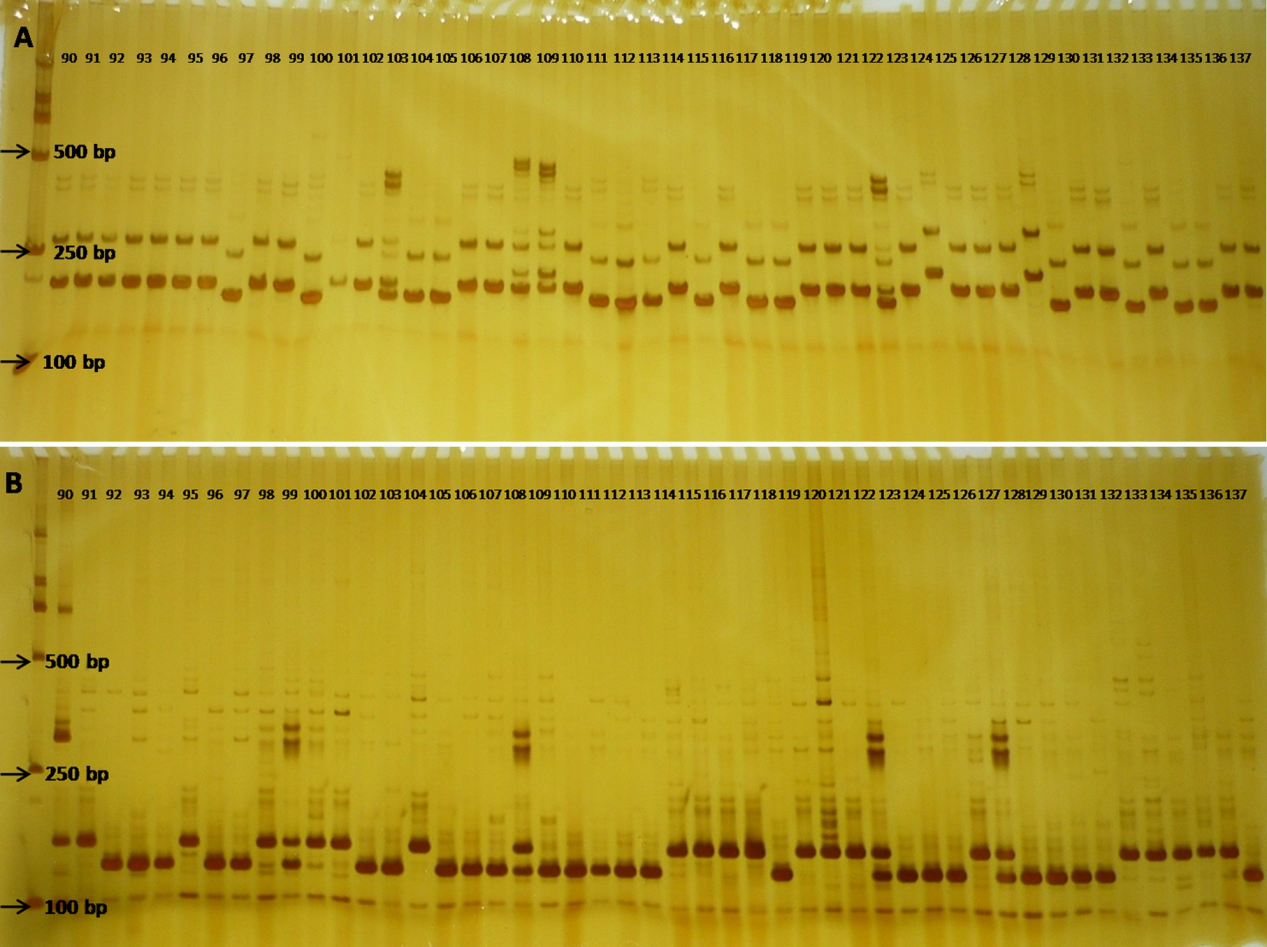
Gel pictures display profile results of SSR markers (A, Ol11H02) and (B, Ol11G11) amplified from some cauliflower inbred lines.

All 43 prescreened SSR primers were used to detect the molecular variance among the 165 cauliflower inbred lines (Fig 1, S2 Table). The parameter values of number of alleles (Na), effective number of alleles (Ne), Shannon’s Information index (I), and polymorphism information content (PIC) per locus were used to estimate genetic diversity, which are listed in S2 Table. The number of alleles (Na) per locus varied from 2 to 6 with a mean value of 2.581, whereas the effective number of alleles (Ne) per locus ranged from 1.019 to 3.200 with an average value of 1.599. The average value of Shannon’s Information index (I) was 0.517, with a range of values from 1.215 to 0.054. The average value of polymorphism information content (PIC) was 0.316, with a range of values from 0.019 to 0.687.

### Genetic diversity and relationships assessed by SSR markers

**Fig 2:**
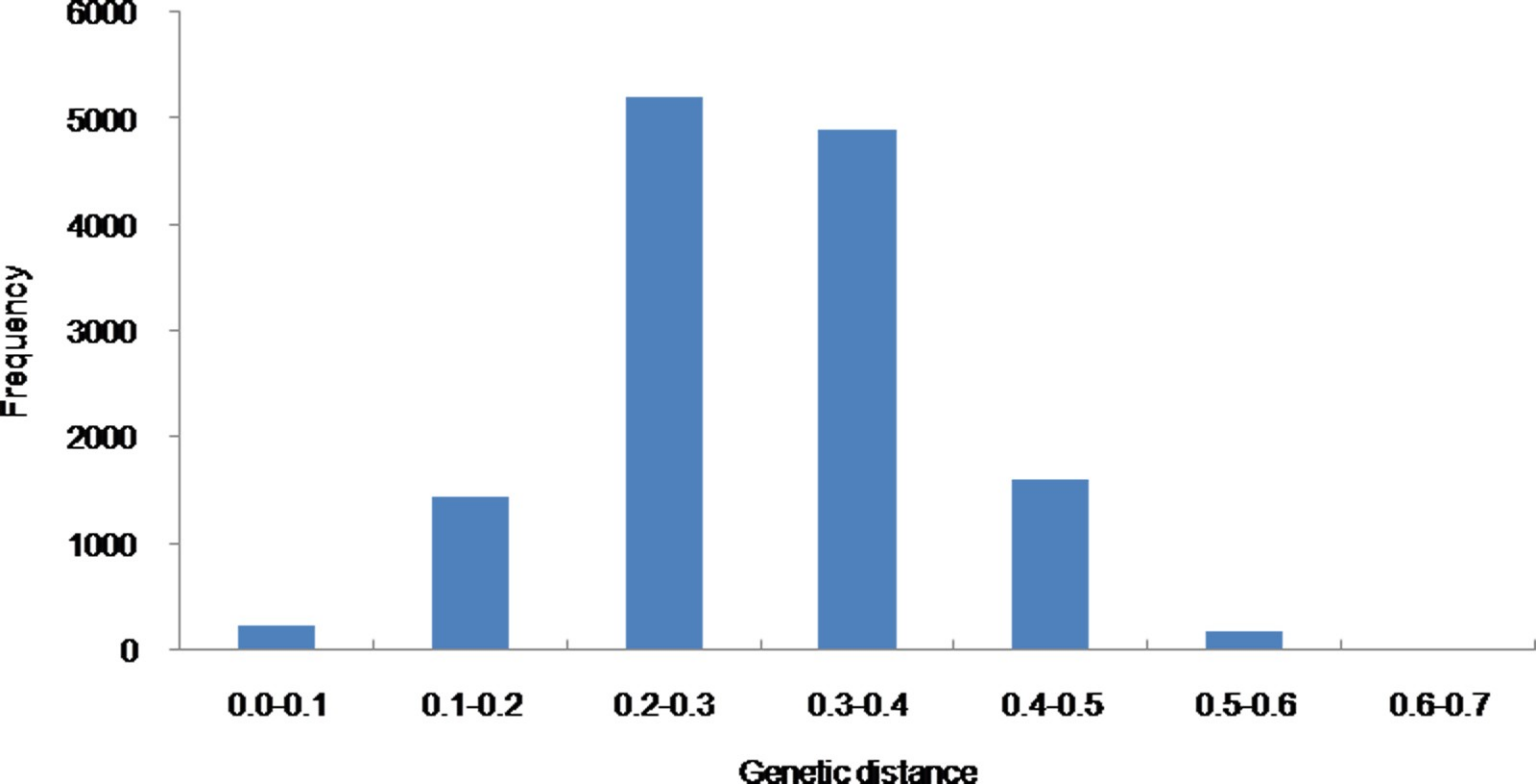
Frequency of pair wise genetic distances in the 165 cauliflower inbred lines.

Nei’s (1972) genetic distance matrix data were calculated for all inbred lines on the basis of the SSR markers. Fig 2 shows the frequency of pair wise genetic distances among the 165 cauliflower inbred lines. The average of pair wise genetic distance values was 0.30. The greatest pair wise genetic distance was 0.67 between WX100 and QGSH65, whereas the smallest pair wise genetic distance was zero between the following: TB80 and YG40A, JZ80 and WX80, AYXF60 and WX80, XLH65 and WX80, SF120 and BY100, YDJG120 and XLH65, YDJG120 and WX80, TDBX120 and XMSH120, R8 and R9.

**Fig 3:**
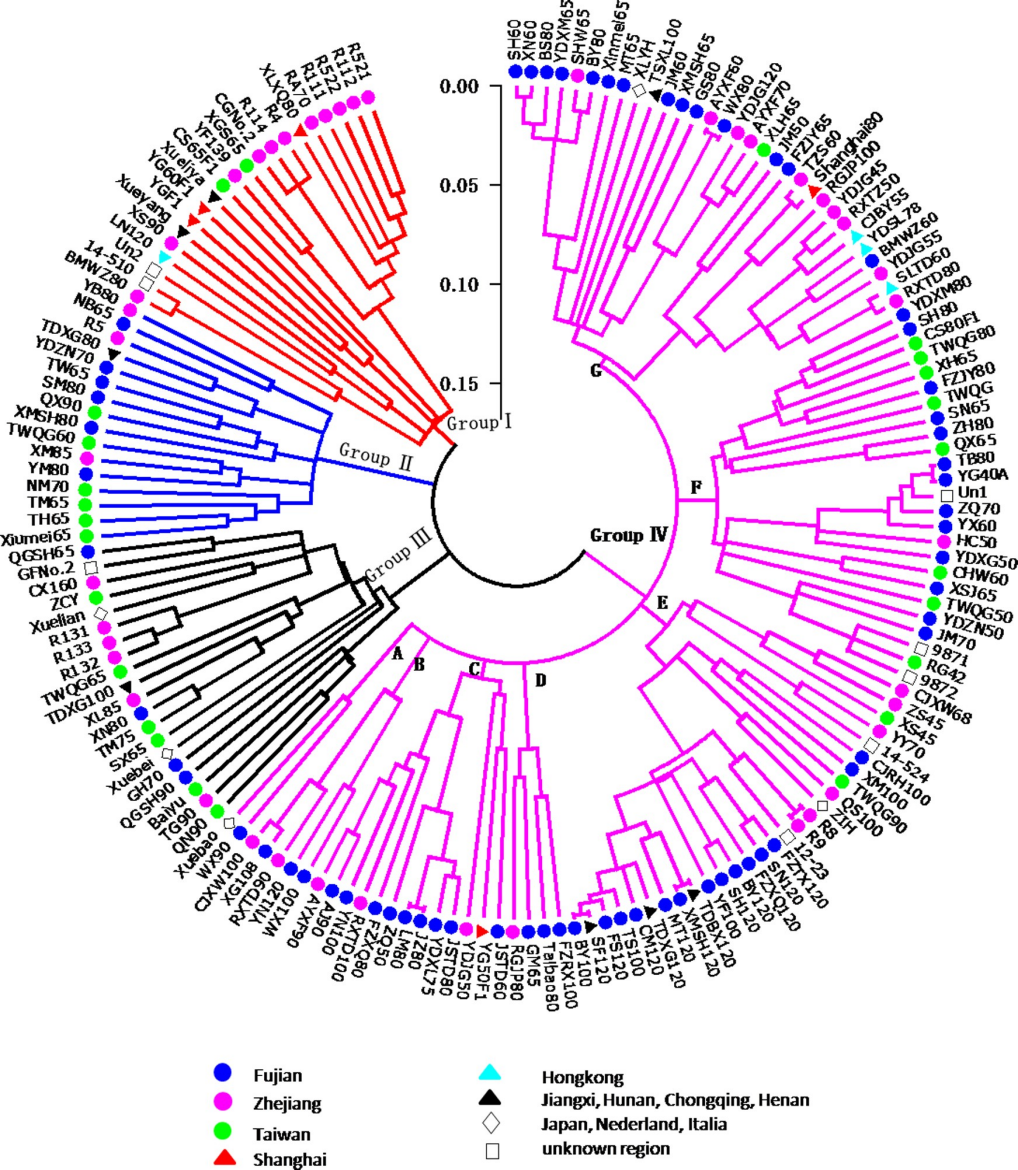
Dendrogram constructed with a neighbor-joining (NJ) clustering algorithm from the genetic distance matrix data calculated from 43 SSR markers among the 165 cauliflower inbred lines.

To estimate the genetic diversity and relationships among these inbred lines, cluster analysis was performed based on the genetic distance matrix data estimated using SSR markers. The dendrogram constructed by neighbor-joining (NJ) cluster analysis showed that the clustering pattern was complex (Fig 3). All 165 inbred lines were divided into four groups, Ⅰ, Ⅱ, Ⅲ, and Ⅳ, containing 16–108 lines per group. Group Ⅰ consisted of 14 late-maturing lines and 7 intermediate-maturing lines primarily derived from Zhejiang, Shanghai, Taiwan, Henan and Hongkong. Group Ⅱ included 16 lines derived from Taiwan, Fujian, Zhejiang and Jiangxi, with 13 of the lines intermediate-maturing. Group Ⅲ contained 15 intermediate-maturing lines and 5 late-maturing lines originating from Taiwan, Fujian, Zhejiang, Jiangxi, Japan and Nederland. Group Ⅳ comprised the remaining 108 lines and was subdivided into seven subgroups marked with symbols from A to G (Fig 3). The lines WX90 and Xuebao derived from Fujian and Japan, respectively, were assigned to subgroup A. Five late-maturing lines were assigned to subgroup B. Thirteen lines were assigned to subgroup C, 4 to subgroup D, 28 to subgroup E, 25 to subgroup F, and 31 to subgroup G. In general, the inbred lines in the subgroups were also complex with maturity and geography. For example, the subgroup G concluded 15 early-maturing lines, 12 intermediate-maturing lines and 4 late-maturing lines originating from Fujian, Zhejiang, Taiwan, Hongkong, Shanghai, Chongqing and Japan. The cluster also presented high genetic similarity between AYXF60 and WX80, TB80 and YG40A, R8 and R9, SF120 and BY100, and JZ80 and LM80 (Fig 3).

**Fig 4:**
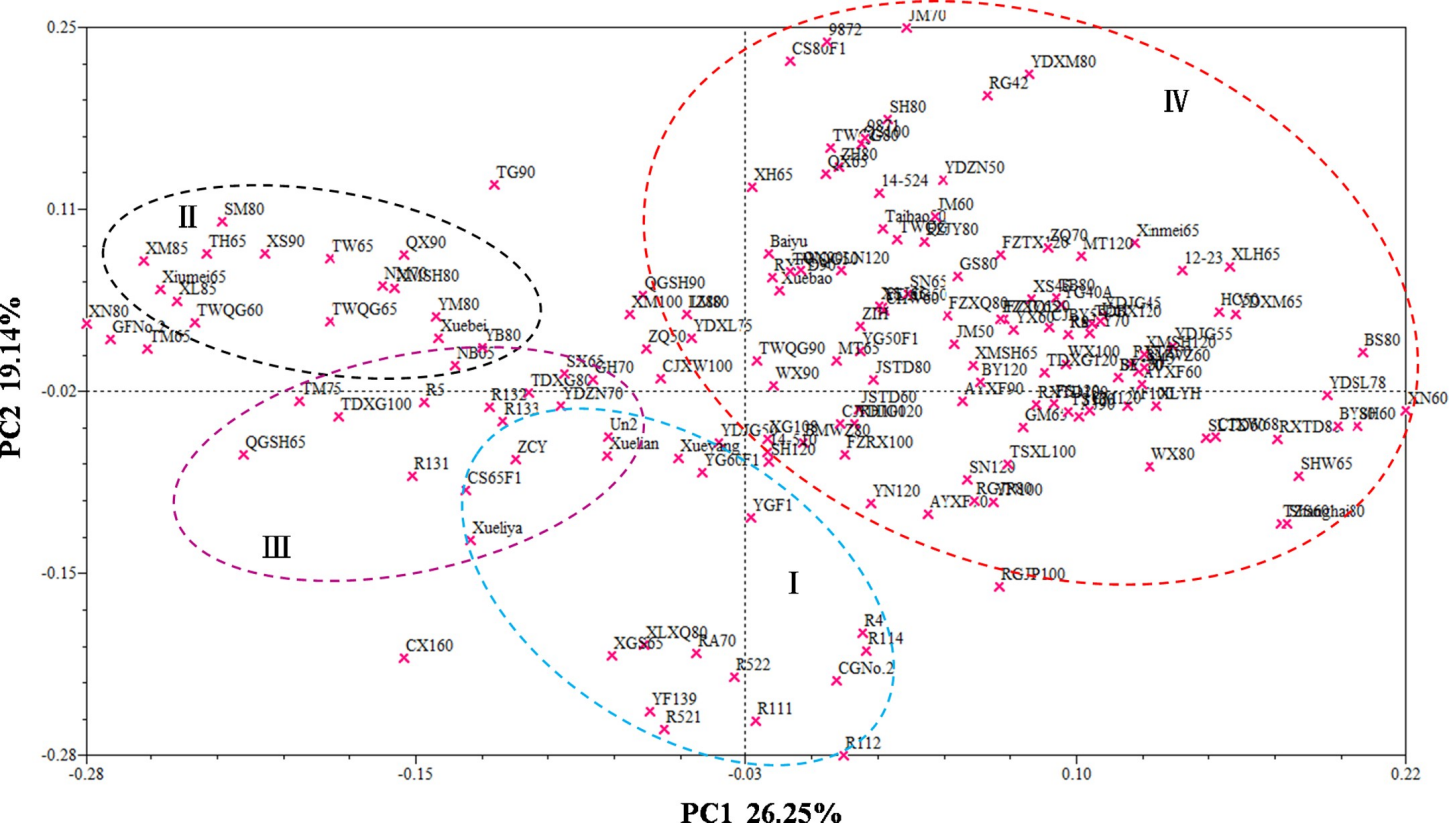
Associations among the 165 cauliflower inbred lines revealed by principal coordinate analysis (PCA) performed on Nei’s genetic distance matrix data calculated from 43 SSR markers.

Principle coordinate analysis (PCA) was chosen to complement the cluster analysis information and genetic relationships [34]. Associations among the 165 inbred lines were examined with a PCA of the genetic distance matrix data from the 43 SSR markers. PCA revealed similar grouping of the inbred lines compared with that of the cluster analysis (Fig 4). This multivariate approach determined that the first two principle components (PC) explained 45.39% of the total variation (26.25% for PC1 and 19.14% for PC2). Genotypes clustered primarily in group Ⅱ were positioned on the positive of the PC2 axis, whereas the inbred lines assigned to group Ⅰ were predominantly on the negative side. Genotypes from group Ⅳ were primarily positioned on the right side of the plot, whereas inbred lines from group Ⅲ were primarily located at the left plot side between groups Ⅰ and Ⅱ.

**Fig 5:**
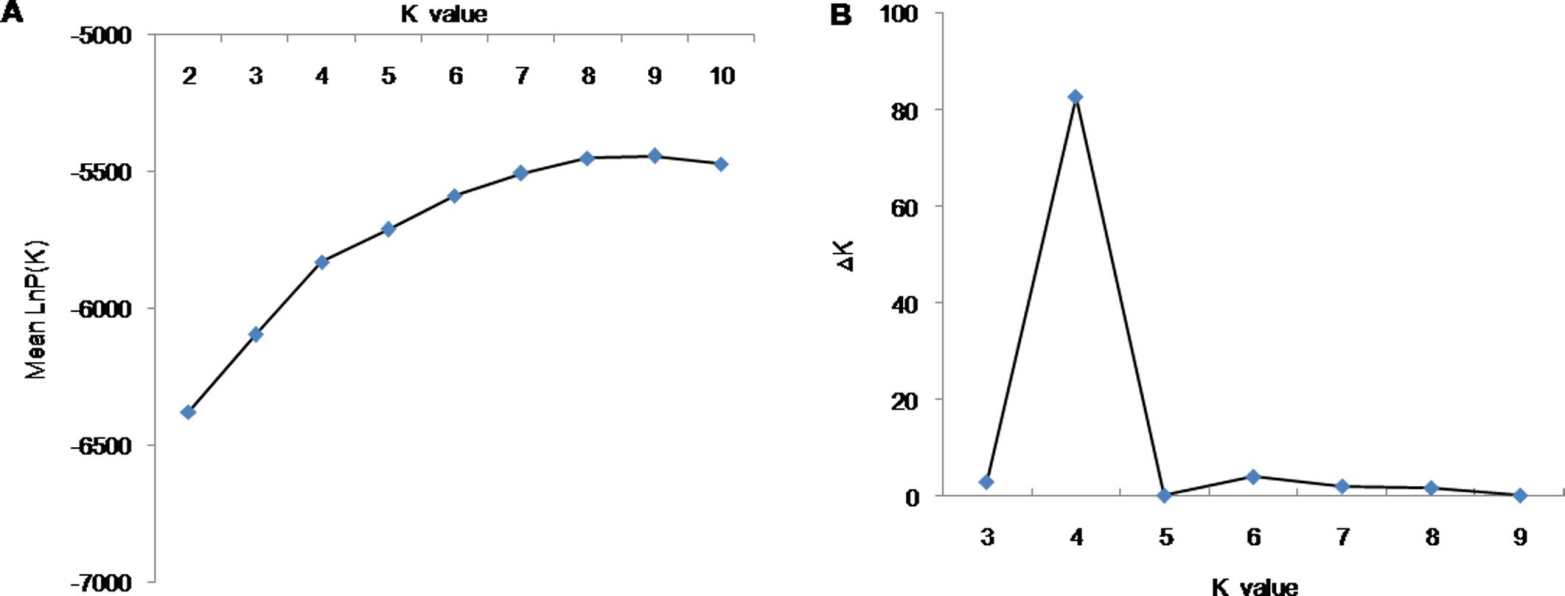
The estimated mean log-likelihood of K values (A) and ΔK values (B).

**Fig 6:**
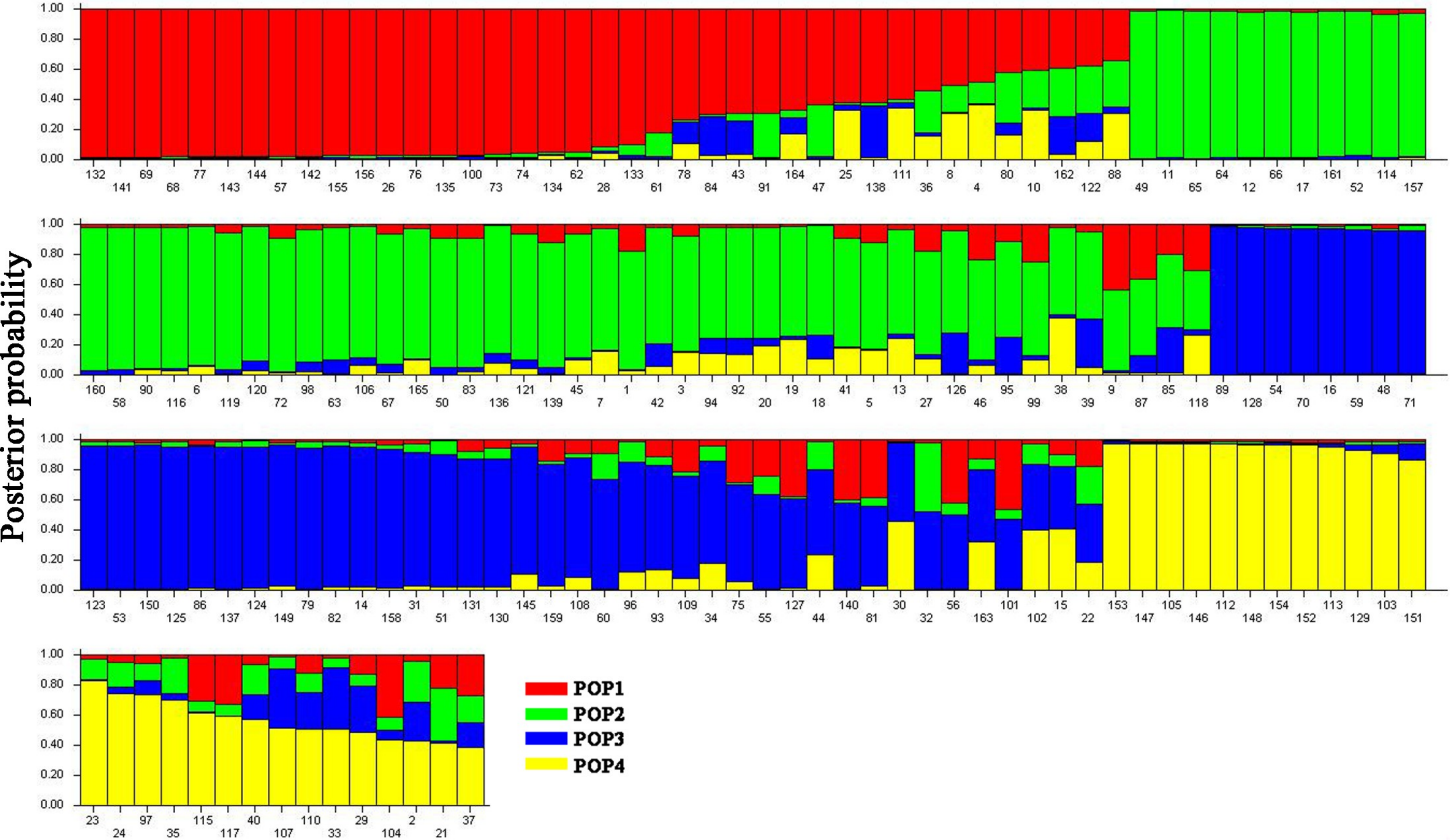
Population structure of the 165 cauliflower inbred lines based on 43 SSR markers.

Structure analysis using the 43 SSR markers revealed that the LnP(D) (log-likelihood) increased with the model parameter K value (Fig 5A). The statistic ΔK was further used to determine a suitable K value (population number). A sharp peak with a maximum value of ΔK was obtained at K = 4 (Fig 5B). This finding indicated that the population of the 165 cauliflower inbred lines we studied was a mixed population consisting of four subpopulations, viz., from POP 1 to POP 4 (Fig 6). In total, 39 accessions (23.6%) were assigned into POP 1, which contained mostly accessions from Fujian, Zhejiang, Hunan, Hongkong, Taiwan, Jiangxi, Italia, and Japan, among others. POP 2 contained 53 accessions, which mostly were from Fujian, Zhejiang, Taiwan, and Hongkong, among others. POP 3 contained 46 accessions, which mostly were from Fujian, Zhejiang, Taiwan, Jiangxi, Japan, and Nederland, among others. POP 4 contained 27 accessions, which were from Zhejiang, Fujian, Shanghai, Taiwan, Chongqing, and Henan, among others. Additionally, almost all subpopulations contained different curd maturity and curd solidity cauliflower inbred lines. These results indicated that the genetic structure classification pattern of most inbred lines was not consistent with their curd maturity, curd solidity or geographic origins.

### Comparison of genetic diversity among different populations

The genetic diversity of the four groups (Ⅰ, Ⅱ, Ⅲ, and Ⅳ) evaluated by the SSR markers is shown in Table 1. Among the four groups, group Ⅰ had the highest genetic diversity based on effective number of alleles (Ne = 1.5277), Shannon’s Information index (I = 0.4941), and polymorphism information content (PIC = 0.2972), whereas group Ⅱ exhibited the lowest genetic diversity based on values of effective number of alleles (Ne = 1.4134), Shannon’s Information index (I = 0.3671), and polymorphism information content (PIC = 0.2319). The genetic diversity of the four groups was ordered as group >group Ⅳ>group Ⅲ>group Ⅱ.

**Table 1:** The number of alleles (Na), effective number of alleles (Ne), Shannon’s Information index (I) and polymorphism information content (PIC) of four groups estimated by 43 SSR markers.

Group	Na	Ne	I	PIC	Origin and number of genotypes by regions	Total number of genotypes
**Ⅰ**	2.3721	1.5277	0.4941	0.2972	Zhejiang (11), Shanghai(3), Henan (2), Taiwan (2), Hongkong (1), unknown (2)	21
**Ⅱ**	1.8837	1.4134	0.3671	0.2319	Taiwan (6), Fujian (6), Zhejiang (3), Jiangxi (1)	16
**Ⅲ**	2.2558	1.4447	0.4354	0.2634	Zhejiang (6), Taiwan (6), Fujian (4), Jiangxi (1), Japan (1), Nederland (1), unknown (1)	20
**Ⅳ**	2.4884	1.5245	0.4596	0.2804	Fujian (56), Zhejiang (24), Taiwan (11), Hongkong (3), Shanghai (2), Hunan (2), Jiangxi (1), Chongqing (1), Japan (2), Italia (1), unknown (5)	108

The genetic diversity of different maturity populations assessed by the SSR markers is presented in Table 2. The intermediate maturity population exhibited the highest genetic diversity based on effective number of alleles (Ne = 1.5901), Shannon’s Information index (I = 0.5019), and polymorphism information content (PIC = 0.3101). The late maturity population had the second highest genetic diversity based on effective number of alleles (Ne = 1.5530), Shannon’s Information index (I = 0.5031), and polymorphism information content (PIC = 0.3067). Compared with the other population types, the early maturity population displayed obviously lower genetic diversity based on effective number of alleles (Ne = 1.5321), Shannon’s Information index (I = 0.4345), and polymorphism information content (PIC = 0.2750).

**Table 2:** Then number of alleles (Na), effective number of alleles (Ne), Shannon’s Information index (I) and polymorphism information content (PIC) of different maturity populations estimated by 43 SSR markers.

Population	Na	Ne	I	PIC	Groups and number of genotypes	Total number of genotypes
**Early maturity**	2.0233	1.5321	0.4345	0.2750	Ⅱ(2), Ⅳ(31)	33
**Intermediate maturity**	2.4419	1.5901	0.5019	0.3101	Ⅰ(6), Ⅱ(12), Ⅲ(15), Ⅳ(43)	76
**Late maturity**	2.4419	1.5530	0.5031	0.3067	Ⅰ(15), Ⅱ(2), Ⅲ(5), Ⅳ(34)	56

The genetic diversity of different curd solidity populations assessed by the SSR markers is shown in Table 3. Among the three curd solidity populations, the loose curd population showed the highest genetic diversity with Ne = 1.6004, I = 0.5034, and PIC = 0.3123, respectively. The compact curd population had the second highest genetic diversity with Ne = 1.5550, I = 0.5016, and PIC = 0.3070, whereas the intermediated curd population exhibited the lowest genetic diversity with Ne = 1.5315, I = 0.4708, and PIC = 0.2855.

**Table 3:** The number of alleles (Na), effective number of alleles (Ne), Shannon’s Information index (I) and polymorphism information content (PIC) of different curd solidity populations estimated by 43 SSR markers.

Population	Na	Ne	I	PIC	Groups and number of genotypes	Total number of genotypes
**Compact curd**	2.5349	1.5550	0.5016	0.3070	Ⅰ(15), Ⅲ(4), Ⅳ(49)	68
**Intermediate curd**	2.3721	1.5315	0.4708	0.2855	Ⅰ(2), Ⅱ(5), Ⅲ(8), Ⅳ(29)	44
**Loose curd**	2.3256	1.6004	0.5034	0.3123	Ⅰ(4), Ⅱ(11), Ⅲ(8), Ⅳ(30)	53

Fujian, Zhejiang and Taiwan are the areas in which early cauliflower breeding programs were conducted in China. To clearly understand the genetic variation in cauliflower inbred lines derived from these areas, the genetic diversity was also estimated by the SSR data (Table 4). The inbred lines derived from Taiwan had relatively high genetic diversity based on effective number of alleles (Ne = 1.5891), Shannon’s Information index (I = 0.4817), and polymorphism information content (PIC = 0.2997), whereas the inbred lines derived from Fujian and Zhejiang showed almost the same relatively low genetic diversity with Ne = 1.5619, I = 0.4748, and PIC = 0.2944 and Ne = 1.5459, I = 0.4734, and PIC = 0.2944, respectively.

**Table 4:** The number of alleles (Na), effective number of alleles (Ne), Shannon’s Information index (I) and polymorphism information content (PIC) of genotypes from Fujian, Zhejiang and Taiwan estimated by 43 SSR markers.

Origin	Na	Ne	I	PIC	Total number of genotypes
**Fujian**	2.3721	1.5619	0.4748	0.2944	66
**Zhejiang**	2.3023	1.5459	0.4734	0.2944	44
**Taiwan**	2.1860	1.5891	0.4817	0.2997	25

## Discussion

Molecular marker types and population size are the factors that influence the evaluation of genetic diversity and relationships. SSR markers play an important role in establishment of genetic diversity and relationships because they are reproducible, polymorphic, codominant and abundant in plant genomes [35–36]. Forty-three SSR markers produced 111 alleles among the 165 cauliflower inbred lines, and the mean values of the number of alleles (Na), effective number of alleles (Ne), Shannon’s Information index (I), and polymorphism information content (PIC) were 2.581, 1.599, 0.517, and 0.316, respectively (S2 Table). The mean PIC value (0.316) was higher than the 0.22 for five cauliflower cultivars estimated by Izzah et al. [15] and lower than the 0.57 for four cauliflower accessions estimated by Iniguez-Luy et al. [37] and the 0.60 for fifty-seven *Brassica oleracea* genotypes (including 51 cauliflower varieties) estimated by Zhao et al. [3]. The relatively low PIC values (average 0.316) revealed the relatively narrow genetic diversity observed in these cauliflower inbred lines, which might be related to the high frequency of artificial-oriented selecting, limited polymorphism of the SSR markers, or low genetic diversity in the breeding materials of companies.

Genetic distance estimates can be used as the basis for effective utilization of the inbred lines with diverse genetic backgrounds for hybridization in breeding programs [38]. The genetic distance between all pairs of the 165 inbred lines varied from 0 to 0.67 with an average of 0.30. Girke et al. estimated similar genetic distance (0.21–0.36) in *Brassica napus* L [39]. Most of these inbred lines are currently used in our hybridization breeding program as parental lines, but the efficiency of utilization remains low because of their low genetic distance. We also found that the genetic distance of nine pair wise lines (TB80 and YG40A, JZ80 and WX80, AYXF60 and WX80, XLH65 and WX80, SF120 and BY100, YDJG120 and XLH65, YDJG120 and WX80, TDBX120 and XMSH120, R8 and R9) was zero. These pair wise inbred lines likely had very similar genetic background, making it difficult to distinguish them using these SSR markers. Our cluster analysis produced a similar observation, and the few inbred lines that could not be differentiated, TB80 and YG40A, R8 and R9, JZ80 and LM80 (Fig 3), were perhaps very closely related because they derived from the same geographic regions. Therefore, hybridization breeding programs in cauliflower should utilize the inbred lines with larger genetic distance to maximize the expression of heterosis [5].

Cluster analysis and principle coordinate analysis are helpful for breeders to understand the genetic diversity and relationships of parents to obtain desirable hybridization combinations [3, 34, 38]. In this study, the 165 cauliflower inbred lines were divided into four major groups by SSR markers (Fig 3), and the PCA also exhibited similar groupings. Different groups exhibited various levels of genetic diversity (group Ⅰ>group Ⅳ>group Ⅲ>group Ⅱ). We found that the clustering patterns of most cauliflower inbred lines were clearly not consistent with the geographic locations from which the varieties were collected (Fig 3 and Table 1). For example, the two accessions (Xuebao and Xuebei) derived from Japanese compact-curd cultivars were divided into different groups (group Ⅲ and Ⅳ, respectively). Additionally, most of the inbred lines derived from Taiwan, Fujian and Zhejiang were clustered together within all four groups (Fig 3 and Table 1), which might be explained by the high frequency of interchange of cultivars or artificial-oriented selection resulting in greater genetic distance than geographical distance in cauliflowers. Similar results are reported in cauliflower and other species [9, 14, 21, 40].

Population structure analysis showed that four subpopulations occurred in the 165 cauliflower inbred lines using the model-based method STRUCTURE. We also found that the structure analysis did not reveal a clear pattern of classification of most cauliflower inbred lines according to their curd maturity, curd solidity or geographic origins. For example, the cauliflower inbred lines derived from Zhejiang and Fujian were classified to all four subpopulations. Similar results were also observed in the cluster analysis (Fig 3 and Table 1). We also found some discrepancies between structure analysis and cluster analysis, which might be because cluster analysis assigned a fixed branch position to each line, whereas structure analysis resulted in a subpopulation membership percentage to assign individuals to groups [35].

Maturity is routinely regarded as the most reliable index for classifying cauliflower cultivars in previous study [41]. However, we found that most cauliflower inbred lines did not cluster tightly according to their maturity (Fig 3 and Table 2). For example, both group Ⅱ and Ⅳ included early-maturing, intermediate-maturing and late-maturing lines together. Interestingly, some early-maturing accessions (e.g., YG60F1 and XGS65 derived from Shanghai and Taiwan, respectively) after continuous self-selecting for several years are currently used in our breeding program as late-maturing resources. These results suggested that some introgression occurred into the gene pool of different maturity cauliflower varieties.

Curd solidity is one important trait that is usually considered by breeders in cauliflower breeding. Breeders usually assess genetic relationships using curd solidity as a simple index for classifying cauliflower germplasms. However, we also found that most cauliflower inbred lines did not cluster tightly according to their curd solidity (Table 3). Thus, in cauliflower breeding, inbred lines with different curd solidity should be utilized comprehensively to obtain desirable hybridization combinations.

Cauliflower has narrow genetic variation based on some previous studies [2–3, 21]. We found that the inbred lines derived from the primarily areas for cauliflower breeding in China (Fujian, Zhejiang and Taiwan) also showed relatively low genetic diversity (Table 4). Thus, we suggest that the genetic diversity of these lines should be increased by pyramiding the valuable genes that exist among different types [3]. Additionally, more polymorphic SSR markers should be used to assess and provide reliable information on genetic diversity for utilization in cauliflower breeding programs.

## Conclusions

The 165 cauliflower inbred lines presented relatively narrow genetic diversity assessed by the 43 SSR markers, and were clustered into four primarily categories by cluster analysis and structure analysis. The clustering patterns of the main inbred lines were clearly not consistent with their curd maturity, curd solidity or geographic origins. Therefore, we suggest that valuable genes among different types of inbred lines be pyramided to increase genetic diversity to obtain desirable hybridization combinations in future cauliflower breeding programs.

## Supporting information

S1 Table The cauliflower inbred lines evaluated for SSR polymorphisms in this study. * Maturity was expressed as the days from transplant to harvest when the inbred lines were cultivated in autumn in Zhejiang, China. Early maturity (<70d), Intermediate maturity(≥70d and<100d), Late maturity(≥100d).
(DOCX)

S2 Table The sequences, annealing temperature, number of alleles (Na), effective number of alleles (Ne), Shannon’s Information index (I) and polymorphism information content (PIC) per locus of the polymorphic SSR markers. (DOCX)

S3 Table Nei’s (1972) genetic distance matrix data calculated for the 165 cauliflower inbred lines on the basis of the SSR markers. (TXT)

S4 Table The LnP(D) (log-likelihood) function value calculated by the STRUCTURE program for determining a suitable K value. (DOCX)
