# Supplementary material for: The genetic diversity and relationships of cauliflower (Brassica oleracea var. botrytis) inbred lines assessed by using SSR markers
Source: PLoS One. 2018 Dec 6;13(12):e0208551. doi: 10.1371/journal.pone.0208551 (PMC6283626; doi:10.1371/journal.pone.0208551)
Supplement: S1 Table — * Maturity was expressed as the days from transplant to harvest when the inbred lines were cultivated in autumn in Zhejiang, China. Early maturity (<70d), Intermediate maturity(≥70d and<100d), Late maturity(≥100d). (DOCX) [file pone.0208551.s001.docx]

**S1 Table. The cauliflower inbred lines evaluated for SSR polymorphisms in this study.**

| No. | Name of original accession | Origin of accession | Year of  collection | Name of  inbred line | Curd solidity of  inbred line | *Maturity of  inbred line |
| --- | --- | --- | --- | --- | --- | --- |
| 1 | Summer Snow (Xiasheng45) | Begreen Seed Co., LTD. (Taiwan, China) | 2000 | XS45 | Compact | Early |
| 2 | Yidaijinguang50 | Wenzhou Dragon-Brand Vegetable Seed Co., LTD. (Zhejiang, China) | 2000 | YDJG50 | Compact | Early |
| 3 | Yidaijinguang55 | Wenzhou Dragon-Brand Vegetable Seed Co., LTD. (Zhejiang, China) | 2000 | YDJG55 | Compact | Early |
| 4 | Shenlongteda60 | Hongkong Sky Dragon Seedling Limited. (Hongkong, China) | Unknown | SLTD60 | Compact | Early |
| 5 | Yidaishenliang78 | Hongkong Sky Dragon Seedling Limited. (Hongkong, China) | 2000 | YDSL78 | Compact | Intermediate |
| 6 | Yidaijinguang45 | Wenzhou Dragon-Brand Vegetable Seed Co., LTD. (Zhejiang, China) | 2000 | YDJG45 | Compact | Intermediate |
| 7 | Ruixuetezao50 | Wenzhou Vegetable Seed Corporation. (Zhejiang, China) | 2000 | RXTZ50 | Loose | Early |
| 8 | Ruixueteda80 | Wenzhou Sanjiao Seed Co., LTD. (Zhejiang, China) | 2000 | RXTD80 | Compact | Intermediate |
| 9 | Yuanye70 | 27°46'N, 120°27'E. (Wenzhou Ruian Mayu, Zhejiang, China) | Unknown | YY70 | Compact | Intermediate |
| 10 | Chaojixuewang68 | Wenzhou Dragon-Brand Vegetable Seed Co., LTD. (Zhejiang, China) | 2000 | CJXW68 | Compact | Intermediate |
| 11 | Yidaizhunong50 | Wenxing Vegetable Seed Co., LTD. (Fujian, China) | 2003 | YDZN50 | Intermediate | Early |
| 12 | Tianbian80 | Xiamen Gaomian Vegetable Seed Co., LTD. (Fujian, China) | 2003 | TB80 | Compact | Intermediate |
| 13 | Jinshengteda80 | Xiamen Maxiang Vegetable Seed Co., LTD. (Fujian, China) | 2003 | JSTD80 | Compact | Early |
| 14 | Tedaxueguan100 | Shangrao Yinong Agricultural Development Co., LTD. (Jiangxi, China) | 2003 | TDXG100 | Intermediate | Intermediate |
| 15 | Xueguan108 | Wenxing Vegetable Seed Co., LTD. (Fujian, China) | 2003 | XG108 | Intermediate | Late |
| 16 | Gaofu No.2 | Unknown | Unknown | GFNo.2 | Loose | Intermediate |
| 17 | Yuguan40A | Xiamen Gaomian Vegetable Seed Co., LTD. (Fujian, China) | 2003 | YG40A | Compact | Intermediate |
| 18 | Zaoqiu50 | Xiamen Tonganyouli Vegetable Seed station. (Fujian, China) | 2003 | ZQ50 | Compact | Early |
| 19 | Jinshengteda60 | Xiamen Maxiang Vegetable Seed Co., LTD. (Fujian, China) | 2003 | JSTD60 | Compact | Intermediate |
| 20 | Yidaixueling75 (704) | Xiamen Gaomian Vegetable Seed Co., LTD. (Fujian, China) | 2003 | YDXL75 | Compact | Early |
| 21 | Aijiao90 | Xiamen Gaomian Vegetable Seed Co., LTD. (Fujian, China) | 2003 | AJ90 | Compact | Intermediate |
| 22 | Wenxing90 | Wenxing Vegetable Seed Co., LTD. (Fujian, China) | 2003 | WX90 | Compact | Intermediate |
| 23 | Yinnong100 (Xuezhen608) | Xiamen Yinnong Seed Co., LTD. (Fujian, China) | 2003 | YN100 | Compact | Late |
| 24 | Wenxing100 | Wenxing Vegetable Seed Co., LTD. (Fujian, China) | 2003 | WX100 | Compact | Late |
| 25 | Fuzhourongxuan100 | Fuzhou Seed Corporation. (Fujian, China) | 2003 | FZRX100 | Loose | Intermediate |
| 26 | Fuzhoutexuan120 | Fujian Gaoda Seed Co., LTD. (Fujian, China) | 2003 | FZTX120 | Intermediate | Intermediate |
| 27 | Fuzhoutedaxueqiu80 | Fuzhou Seed Corporation. (Fujian, China) | 2003 | FZXQ80 | Compact | Intermediate |
| 28 | Shengnong120 | Fuzhou Shengxin Seed Co., LTD. (Fujian, China) | 2003 | SN120 | Intermediate | Late |
| 29 | Yinguan F1 | Shanghai Changzheng Vegetable Seed Co., LTD. (Shanghai, China) | 2009 | YGF1 | Compact | Late |
| 30 | Chunxue160 | Ruian South Vegetable Seed Co., LTD. (Zhejiang, China) | 2009 | CX160 | Compact | Late |
| 31 | Qinggengsonghua65 | Fuzhou Rongzhang Seed Co., LTD. (Fujian, China) | 2009 | QGSH65 | Intermediate | Late |
| 32 | Qinggengsonghua90 | Fuzhou Rongzhang Seed Co., LTD. (Fujian, China) | 2009 | QGSH90 | Loose | Intermediate |
| 33 | Yinguan60 F1 | Shanghai Changzheng Vegetable Seed Co., LTD. (Shanghai, China) | 2009 | YG60F1 | Loose | Late |
| 34 | Chaojixuewang100 | Wenzhou Dragon-Brand Vegetable Seed Co., LTD. (Zhejiang, China) | 2000 | CJXW100 | Compact | Late |
| 35 | Yidaijinguang120 | Wenzhou Dragon-Brand Vegetable Seed Co., LTD. (Zhejiang, China) | 2000 | YDJG120 | Intermediate | Late |
| 36 | Zaosheng45 | Yueqing Yefeng Seed Co., LTD. (Zhejiang, China) | 2005 | ZS45 | Compact | Early |
| 37 | Anyangxuefeng70 | Ruian Anyang Vegetable Seed Co., LTD. (Zhejiang, China) | 2005 | AYXF70 | Compact | Early |
| 38 | Anyangxuefeng90 | Ruian Anyang Vegetable Seed Co., LTD. (Zhejiang, China) | 2006 | AYXF90 | Compact | Intermediate |
| 39 | Ruixueteda90 | Wenzhou Sanjiao Seed Co., LTD. (Zhejiang, China) | 1999 | RXTD90 | Intermediate | Late |
| 40 | Baimawangzi80 | Wenzhou Sanjiao Seed Co., LTD. (Zhejiang, China) | 2003 | BMWZ80 | Compact | Intermediate |
| 41 | Jiamei50 | Wenxing Vegetable Seed Co., LTD. (Fujian, China) | 2007 | JM50 | Loose | Intermediate |
| 42 | Chunhuawang60 | Chinglong Seed Co., LTD. (Taiwan, China) | 2008 | CHW60 | Loose | Intermediate |
| 43 | Gaomei65 (A300) | Xiamen Gaomian Vegetable Seed Co., LTD. (Fujian, China) | 2003 | GM65 | Loose | Late |
| 44 | Nongbao65 | Fujian Nongjia Seed Co., LTD. (Fujian, China) | 2003 | NB65 | Intermediate | Late |
| 45 | Fuzhoujianye65 | Xiamen Huadi Vegetable Seed Co., LTD. (Fujian, China) | Unknown | FZJY65 | Intermediate | Early |
| 46 | Xiamensonghua65 | Xiamen Huadi Vegetable Seed Co., LTD. (Fujian, China) | 2007 | XMSH65 | Loose | Early |
| 47 | Xinmei65 | Xiamen Tonganyouli Vegetable Seed station. (Fujian, China) | 2007 | Xinmei65 | Loose | Early |
| 48 | Xiumei65 | Besgrow Seed Co., LTD. (Taiwan, China) | 2007 | Xiumei65 | Loose | Early |
| 49 | Yidaixuemei80 | Xiamen Huadi Vegetable Seed Co., LTD. (Fujian, China) | 2003 | YDXM80 | Loose | Intermediate |
| 50 | Taiwanqinggeng80 | Taiwan cauliflower Institute. (Taiwan, China) | 2003 | TWQG80 | Loose | Intermediate |
| 51 | Songmei80 | Xiamen Tonganyouli Vegetable Seed station. (Fujian, China) | 2003 | SM80 | Loose | Intermediate |
| 52 | Songhua80 | Wenxing Vegetable Seed Co., LTD. (Fujian, China) | 2007 | SH80 | Loose | Intermediate |
| 53 | Yamei80 | Xiamen Linxingli Vegetable Seed Co., LTD. (Fujian, China) | 2007 | YM80 | Loose | Intermediate |
| 54 | Qingxiu90 | Besgrow Seed Co., LTD. (Taiwan, China) | 2006 | QX90 | Loose | Late |
| 55 | No.80 White Jade (No.80 Baiyu) | Besgrow Seed Co., LTD. (Taiwan, China) | 2007 | Baiyu | Intermediate | Intermediate |
| 56 | Qingnong90 | Chinglong Seed Co., LTD. (Taiwan, China) | 2008 | QN90 | Loose | Intermediate |
| 57 | Mintai120 | Xiamen Huadi Vegetable Seed Co., LTD. (Fujian, China) | 2003 | MT120 | Loose | Late |
| 58 | Changsheng80 F1 | Changshen Seed Co., LTD. (Taiwan, China) | 2009 | CS80F1 | Loose | Intermediate |
| 59 | Xuemei85 | Zhejiang Sky Good Seeds Co., LTD. (Zhejiang, China) | 2009 | XM85 | Loose | Intermediate |
| 60 | Thailand90 (Taiguo90) | Hangzhou Yinhan Seed Co., LTD. (Zhejiang, China) | 2009 | TG90 | Intermediate | Intermediate |
| 61 | Yidaixuemei65 | Xiamen Huadi Vegetable Seed Co., LTD. (Fujian, China) | 2003 | YDXM65 | Intermediate | Early |
| 62 | Bisheng80 | Xiamen Linxingli Vegetable Seed Co., LTD. (Fujian, China) | 2007 | BS80 | Compact | Early |
| 63 | Xiuhua65 | Besgrow Seed Co., LTD. (Taiwan, China) | 2006 | XH65 | Loose | Intermediate |
| 64 | Jiamei70 | Wenxing Vegetable Seed Co., LTD. (Fujian, China) | 2003 | JM70 | Intermediate | Early |
| 65 | Zaoqiu70 | Xiamen Tonganyouli Vegetable Seed station. (Fujian, China) | 2003 | ZQ70 | Compact | Intermediate |
| 66 | Fuzhoujianye80 | Xiamen Huadi Vegetable Seed Co., LTD. (Fujian, China) | 2003 | FZJY80 | Loose | Intermediate |
| 67 | Gaosong80 (A340) | Xiamen Gaomian Vegetable Seed Co., LTD. (Fujian, China) | 2003 | GS80 | Loose | Intermediate |
| 68 | Tedabaixue120 | Zhuzhou Dayang Seed research Center. (Hunan, China) | 2008 | TDBX120 | Intermediate | Late |
| 69 | xiamensonghua120 | Xiamen Huadi Vegetable Seed Co., LTD. (Fujian, China) | 2003 | XMSH120 | Intermediate | Late |
| 70 | Taiwanqinggeng60 | Taiwan cauliflower Institute. (Taiwan, China) | 2003 | TWQG60 | Intermediate | Intermediate |
| 71 | Nongmei70 | Taiwan Nongbao Seed Co., LTD. (Taiwan, China) | 2003 | NM70 | Loose | Intermediate |
| 72 | Haochi50 | Wenzhou Shouzhi Vegetable Seed Co., LTD. (Zhejiang, China) | 2006 | HC50 | Intermediate | Early |
| 73 | Songhua60 | Wenxing Vegetable Seed Co., LTD. (Fujian, China) | 2007 | SH60 | Loose | Intermediate |
| 74 | Xinong60 | Fuzhou Jinming Seed Co., LTD. (Fujian, China) | 2007 | XN60 | Loose | Intermediate |
| 75 | Changsheng65 F1 | Changshen Seed Co., LTD. (Taiwan, China) | 2008 | CS65F1 | Loose | Late |
| 76 | Yafei100 | Fuzhou Tainong Seed Co., LTD. (Fujian, China) | 2007 | YF100 | Compact | Late |
| 77 | Boya120 | Xiamen Linxingli Vegetable Seed Co., LTD. (Fujian, China) | 2007 | BY120 | Intermediate | Late |
| 78 | Songhua120 | Wenxing Vegetable Seed Co., LTD. (Fujian, China) | 2003 | SH120 | Intermediate | Late |
| 79 | Xueli85 | Zhejiang Sky Good Seeds Co., LTD. (Zhejiang, China) | 2009 | XL85 | Loose | Intermediate |
| 80 | Anyangxuefeng60 | Ruian Anyang Vegetable Seed Co., LTD. (Zhejiang, China) | 2006 | AYXF60 | Compact | Intermediate |
| 81 | Linong120 | Hongkong Linong Seed Co., LTD. (Hongkong, China) | 2008 | LN120 | Compact | Late |
| 82 | Xuesong90 | Ruian South Vegetable Seed Co., LTD. (Zhejiang, China) | 2008 | XS90 | Intermediate | Intermediate |
| 83 | Xinlihe65 | Leadhood Seed Co., LTD. (Taiwan, China) | 2008 | XLH65 | Intermediate | Early |
| 84 | Chaojironghua100 | Fuzhou Rong Vegetable Seed Co., LTD. (Fujian, China) | 2007 | CJRH100 | Intermediate | Late |
| 85 | Taibao80 | Xiamen Xianganyouli Vegetable Seed station. (Fujian, China) | 2007 | Taibao80 | Loose | Intermediate |
| 86 | Taimei75 | Shantou Jinhan Seed Co., LTD.(Seeds producted in Taiwan) | 2009 | TM75 | Loose | Late |
| 87 | Qingsong100 | Zhejiang Sky Good Seeds Co., LTD. (Zhejiang, China) | 2007 | QS100 | Loose | Late |
| 88 | Wenxing80 | Wenxing Vegetable Seed Co., LTD. (Fujian, China) | 2003 | WX80 | Compact | Late |
| 89 | Xinong80 | Fuzhou Jinming Seed Co., LTD. (Fujian, China) | 2007 | XN80 | Loose | Intermediate |
| 90 | Chaojibaiyu55 | Hongkong Sky Dragon Seedling Limited. (Hongkong, China) | 2000 | CJBY55 | Compact | Early |
| 91 | 12-23 | Unknown | Unknown | 12-23 | Loose | Late |
| 92 | Jinzhu80 | Xiamen Jinlv Seed Co., LTD. (Fujian, China) | 2003 | JZ80 | Compact | Intermediate |
| 93 | Yidaizhunong70 | Wenxing Vegetable Seed Co., LTD. (Fujian, China) | 2003 | YDZN70 | Intermediate | Intermediate |
| 94 | Lumei80 (607) | Xiamen Gaomian Vegetable Seed Co., LTD. (Fujian, China) | 2003 | LM80 | Intermediate | Intermediate |
| 95 | Zhanghua80 | Wenxing Vegetable Seed Co., LTD. (Fujian, China) | 2003 | ZH80 | Loose | Intermediate |
| 96 | Tedaxueguan80 | Shangrao Yinong Agricultural Development Co., LTD. (Jiangxi, China) | 2003 | TDXG80 | Loose | Intermediate |
| 97 | Yinnong120 (Dongchun609) | Xiamen Yinnong Seed Co., LTD. (Fujian, China) | 2003 | YN120 | Compact | Late |
| 98 | Yidaixiaguan50 (713) | Xiamen Gaomian Vegetable Seed Co., LTD. (Fujian, China) | 2003 | YDXG50 | Compact | Intermediate |
| 99 | Shengnong65 | Xiamen Huadi Vegetable Seed Co., LTD. (Fujian, China) | 2003 | SN65 | Compact | Intermediate |
| 100 | Tedaxueguan120 | Shangrao Yinong Agricultural Development Co., LTD. (Jiangxi, China) | 2003 | TDXG120 | Loose | Late |
| 101 | Xuebao | Sakata Seed Corporation. (Japan) | 2009 | Xuebao | Intermediate | Late |
| 102 | Xueyang | Zhengzhou Vegetable Institute. (Henan, China) | 2009 | Xueyang | Loose | Intermediate |
| 103 | Xinglvxueqiu80 | Shanghai Xinglv Vegetable Seed Institute. (Shanghai, China) | 2006 | XLXQ80 | Compact | Intermediate |
| 104 | Tianshanxuelian100 | Chongqing Huayu Seed Co., LTD. (Chongqing,China) | 2009 | TSXL100 | Compact | Late |
| 105 | Chenggong No.2 | Zhejiang Qingyi Seed Co., LTD. (Zhejiang, China) | 2009 | CGNo.2 | Compact | Late |
| 106 | Yinguan50 F1 | Shanghai Changzheng Vegetable Seed Co., LTD. (Shanghai, China) | 2009 | YG50F1 | Intermediate | Early |
| 107 | Xueliya | Zhengzhou Vegetable Institute. (Henan, China) | 2009 | Xueliya | Loose | Late |
| 108 | Snow Baby (Xuebei) | Musashino Seed Co., LTD. (Japan) | 2009 | Xuebei | Compact | Intermediate |
| 109 | Xuelian | China Seed Group Corporation (Seeds producted in Nederland) | 2009 | Xuelian | Compact | Late |
| 110 | 14-510 | Unknown | Unknown | 14-510 | Compact | Intermediate |
| 111 | Rengongjiaopei80 | Ruian South Vegetable Seed Co., LTD. (Zhejiang, China) | 2006 | RGJP80 | Compact | Late |
| 112 | Rengongjiaopei100 | Ruian South Vegetable Seed Co., LTD. (Zhejiang, China) | 2006 | RGJP100 | Compact | Intermediate |
| 113 | Yefeng139 | Yueqing Yefeng Seed Co., LTD. (Zhejiang, China) | 2005 | YF139 | Compact | Late |
| 114 | Heat Crown (Reguan42) | Begreen Seed Co., LTD. (Taiwan, China) | 2007 | RG42 | Compact | Early |
| 115 | Tezaosheng60 | Ruian South Vegetable Seed Co., LTD. (Zhejiang, China) | 2006 | TZS60 | Compact | Intermediate |
| 116 | Yingxue60 | Xiamen Huadi Vegetable Seed Co., LTD. (Fujian, China) | 2008 | YX60 | Compact | Early |
| 117 | Shanghai80 | Shanghai Agricultural Science and Technology Seed Co., LTD. (Shanghai, China) | 2004 | Shanghai80 | Compact | Intermediate |
| 118 | Ruixueteda100 | Wenzhou Vegetable Seed Corporation. (Zhejiang, China) | 1999 | RXTD100 | Compact | Late |
| 119 | 14-524 | Unknown | Unknown | 14-524 | Compact | Intermediate |
| 120 | Taiwanqinggeng50 | Taiwan cauliflower Institute. (Taiwan, China) | 2003 | TWQG50 | Loose | Early |
| 121 | Jiamei60 | Wenxing Vegetable Seed Co., LTD. (Fujian, China) | 2003 | JM60 | Loose | Intermediate |
| 122 | Mintai65 (615) | Xiamen Huadi Vegetable Seed Co., LTD. (Fujian, China) | 2007 | MT65 | Intermediate | Early |
| 123 | Taiwan65 | Xiamen Xianganyouli Vegetable Seed station. (Fujian, China) | 2007 | TW65 | Loose | Intermediate |
| 124 | Taihua65 | Xiamen Jinlv Seed Co., LTD.(Original seeds from Taiwan) | 2007 | TH65 | Loose | Intermediate |
| 125 | Taiwanqinggeng65 | Taiwan cauliflower Institute. (Taiwan, China) | 2007 | TWQG65 | Intermediate | Intermediate |
| 126 | Qingxiu65 | Besgrow Seed Co., LTD. (Taiwan, China) | 2007 | QX65 | Loose | Intermediate |
| 127 | Songxue65 | Pan-Asian Seeds Co., LTD. (Taiwan, China) | 2007 | SX65 | Loose | Intermediate |
| 128 | Taimei65 | Taiwan Ta San Seed Co., LTD. (Taiwan, China) | 2008 | TM65 | Loose | Intermediate |
| 129 | Xingui65 | Suntech Seed Co., LTD. (Taiwan, China) | 2008 | XGS65 | Intermediate | Late |
| 130 | Gaohua70 (A320) | Xiamen Gaomian Vegetable Seed Co., LTD. (Fujian, China) | 2003 | GH70 | Loose | Intermediate |
| 131 | Yinbei80 | Wenzhou Sanjiao Seed Co., LTD. (Zhejiang, China) | 2008 | YB80 | Intermediate | Early |
| 132 | Shuangfeng120 | Hunan Yueyang New Vegetable Seed sales department. (Hunan, China) | 2008 | SF120 | Intermediate | Late |
| 133 | Fuzhouxueqiu120 | Fuzhou Rongzhang Vegetable Seed Co., LTD. (Fujian, China) | 2007 | FZXQ120 | Intermediate | Intermediate |
| 134 | Songhuawang65 | Yueqing Zhaofeng seedling Co., LTD. (Zhejiang, China) | 2009 | SHW65 | Loose | Early |
| 135 | Boya80 | Xiamen Linxingli Vegetable Seed Co., LTD. (Fujian, China) | 2007 | BY80 | Loose | Early |
| 136 | Xinshiji65 | Xiamen Jinlv Seed Co., LTD. (Fujian, China) | 2008 | XSJ65 | Loose | Intermediate |
| 137 | Xiamensonghua80 | Xiamen Huadi Vegetable Seed Co., LTD. (Fujian, China) | 2003 | XMSH80 | Loose | Intermediate |
| 138 | Taiwanqinggeng90 | Taiwan cauliflower Institute. (Taiwan, China) | 2007 | TWQG90 | Loose | Intermediate |
| 139 | Taiwanqinggeng | Taiwan cauliflower Institute. (Taiwan, China) | Unknown | TWQG | Intermediate | Early |
| 140 | Ximei100 | Xiamen Xianganyouli Vegetable Seed station. (Fujian, China) | 2007 | XM100 | Loose | Late |
| 141 | Boya100 | Xiamen Linxingli Vegetable Seed Co., LTD. (Fujian, China) | Unknown | BY100 | Compact | Late |
| 142 | Taisong100 | Xiamen Huadi Vegetable Seed Co., LTD. (Fujian, China) | 2008 | TS100 | Intermediate | Late |
| 143 | Fusong120 | Fuzhou Nongyu Seed Shop. (Fujian, China) | 2003 | FS120 | Intermediate | Late |
| 144 | Chunmei120 | Xiamen Xianganyouli Vegetable Seed station. (Fujian, China) | 2008 | CM120 | Compact | Late |
| 145 | Rui5 | 27°46'N, 120°27'E. (Wenzhou Ruian Mayu, Zhejiang, China) | 2008 | R5 | Intermediate | Intermediate |
| 146 | Rui11-1 | 27°46'N, 120°41'E. (Wenzhou Ruian Xincheng, Zhejiang, China) | 2009 | R111 | Compact | Late |
| 147 | Rui11-2 | 27°46'N, 120°41'E. (Wenzhou Ruian Xincheng, Zhejiang, China) | 2009 | R112 | Compact | Intermediate |
| 148 | Rui11-4 | 27°46'N, 120°27'E. (Wenzhou Ruian Mayu, Zhejiang, China) | 2008 | R114 | Compact | Late |
| 149 | Rui131 | 27°46'N, 120°27'E. (Wenzhou Ruian Mayu, Zhejiang, China) | 2008 | R131 | Intermediate | Intermediate |
| 150 | Rui132 | 27°46'N, 120°27'E. (Wenzhou Ruian Mayu, Zhejiang, China) | 2008 | R132 | Intermediate | Intermediate |
| 151 | Ruian70 | 27°46'N, 120°41'E. (Wenzhou Ruian Xincheng, Zhejiang, China) | 2009 | RA70 | Compact | Late |
| 152 | Rui4 | 27°46'N, 120°27'E. (Wenzhou Ruian Mayu, Zhejiang, China) | 2008 | R4 | Compact | Late |
| 153 | Rui521 | 27°46'N, 120°27'E. (Wenzhou Ruian Mayu, Zhejiang, China) | 2008 | R521 | Compact | Late |
| 154 | Rui522 | 27°46'N, 120°27'E. (Wenzhou Ruian Mayu, Zhejiang, China) | 2008 | R522 | Compact | Late |
| 155 | Rui8 | 27°46'N, 120°27'E. (Wenzhou Ruian Mayu, Zhejiang, China) | 2008 | R8 | Intermediate | Late |
| 156 | Rui9 | 27°46'N, 120°27'E. (Wenzhou Ruian Mayu, Zhejiang, China) | 2008 | R9 | Intermediate | Late |
| 157 | Un1 | Unknown | Unknown | Un1 | Intermediate | Intermediate |
| 158 | Rui133 | 27°46'N, 120°27'E. (Wenzhou Ruian Mayu, Zhejiang, China) | 2008 | R133 | Intermediate | Intermediate |
| 159 | Zaochunyu | Taiwan First Seed Co., LTD. (Taiwan, China) | 2010 | ZCY | Compact | Late |
| 160 | 987-1 | Unknown | 2009 | 9871 | Intermediate | Early |
| 161 | 987-2 | Unknown | 2009 | 9872 | Compact | Early |
| 162 | Violetto di Sicilia | Ansime Srl. (Italia) | 2001 | ZIH | Compact | Late |
| 163 | Un2 | Unknown | Unknown | Un2 | Compact | Late |
| 164 | Xuelingyihao | Clover Seed Co., Ltd.(Seeds originally producted in Japan) | 2009 | XLYH | Compact | Late |
| 165 | Baimawangzi60 | Wenzhou Sanjiao Seed Co., LTD. (Zhejiang, China) | 2003 | BMWZ60 | Compact | Early |

* Maturity was expressed as the days from transplant to harvest when the inbred lines were cultivated in autumn in Zhejiang, China. Early maturity (＜70d), Intermediate maturity(≥70d and ＜100d), Late maturity(≥00d).
