# Supplementary material for: The genetic diversity and relationships of cauliflower (Brassica oleracea var. botrytis) inbred lines assessed by using SSR markers
Source: PLoS One. 2018 Dec 6;13(12):e0208551. doi: 10.1371/journal.pone.0208551 (PMC6283626; doi:10.1371/journal.pone.0208551)
Supplement: S2 Table — (DOCX) [file pone.0208551.s002.docx]

**S2 Table. The sequences, annealing temperature, number of alleles (Na), effective number of alleles (Ne), Shannon’s Information index (I) and polymorphism information content (PIC) per locus of the polymorphic SSR markers.**

| Primer name | Primer sequence (5’-3’)  (forward, reverse) | Annealing temperature ( ℃) | Na | Ne | I | PIC |
| --- | --- | --- | --- | --- | --- | --- |
| Ni4B04 | TAGGCGGACTTGTTGATTCC | 55 | 2 | 1.203 | 0.310 | 0.169 |
|  | CATGATTCAGAACAAGGGTGC |  |  |  |  |  |
| Ni4B10 | GTCCTTGAGAAACTCCACCG | 55 | 2 | 1.075 | 0.156 | 0.070 |
|  | CCGATCCCATTTCTAATCCC |  |  |  |  |  |
| Ni4D09 | AAAGGACAAAGAGGAAGGGC | 55 | 2 | 1.318 | 0.405 | 0.241 |
|  | TTGAAATCAAATGAGAGTGACG | |  |  |  |  |
| Ni4F09 | CTGTTATGCAAGGTCATCGC | 55 | 4 | 1.056 | 0.145 | 0.053 |
|  | TGTTCCAGGTGAAGAAACCG |  |  |  |  |  |
| Ol10C05 | GGCTACAAAATGTTTGATAAGCTCT | 55 | 3 | 1.318 | 0.456 | 0.242 |
|  | ACCTGAAAGAGAGGCTACACAT | |  |  |  |  |
| Ol10D03 | GCCAAAGACCTCAAAGATGG | 55 | 2 | 1.669 | 0.591 | 0.401 |
|  | AAGCCACGTGAAGAAAGTCC |  |  |  |  |  |
| Ol10D08 | TCCGAACACTCTAAGTTAGCTCC | 55 | 3 | 2.235 | 0.889 | 0.553 |
|  | GAGCTGTATGTCTCCCGTGC |  |  |  |  |  |
| Ol10E04 | ATACCGATAGTCACGGCTCG | 55 | 2 | 1.137 | 0.238 | 0.120 |
|  | GGTGGCAACGTCAGATTACC |  |  |  |  |  |
| Ol10F07 | ACCTCGTTGCAGGTAAACG | 55 | 2 | 1.482 | 0.507 | 0.325 |
|  | GCCTTGAATCTGATTGCTGC |  |  |  |  |  |
| Ol10G05 | TCAATGCTCTTGTAGTCTTTGACC | 55 | 3 | 2.589 | 1.020 | 0.614 |
|  | AGAATGAGAGCGTGGAGAGG |  |  |  |  |  |
| Ol11G11 | GTTGCGGCGAAACAGAGAAG | 55 | 2 | 1.978 | 0.688 | 0.495 |
|  | GAGTAGGCGATCAAACCGAG |  |  |  |  |  |
| Ol12A04 | TGGGTAAGTAACTGTGGTGGC | 55 | 2 | 1.988 | 0.690 | 0.497 |
|  | AGAGTTCGCATACTCTGGAGC |  |  |  |  |  |
| Ol11H02 | TCTTCAGGGTTTCCAACGAC | 55 | 3 | 2.118 | 0.861 | 0.528 |
|  | AGGCTCCTTCATTTGATCCC |  |  |  |  |  |
| Ol12B12 | GACAAACAGCTCCATCAACG | 55 | 3 | 1.045 | 0.115 | 0.043 |
|  | CACGAACACAATCGGTCAAC |  |  |  |  |  |
| Ol12D01 | CATTCAGCACTCGCTCATGG | 55 | 4 | 3.200 | 1.215 | 0.687 |
|  | TCTCAGGCACTCTTTAAAGC |  |  |  |  |  |
| Ol12D05 | TCCATGACCAACGACAAGGTC | 55 | 2 | 1.723 | 0.610 | 0.420 |
|  | AAGAGGCGACTTCTATTGCG |  |  |  |  |  |
| Ol12E03 | CTTGAAGAGCTTCCGACACC | 55 | 3 | 1.151 | 0.297 | 0.131 |
|  | GACGGCTAACAGTGGTGGAC |  |  |  |  |  |
| Ol12F03 | CCTCGTTGCAGGTAAACGAC | 55 | 2 | 1.576 | 0.552 | 0.365 |
|  | GACTTCCGCAGCTCTCTGTC |  |  |  |  |  |
| Ol13C03 | GATCGGAGATGCGATGAGAG | 58 | 3 | 2.006 | 0.743 | 0.501 |
|  | GCATGCACCAGTGAAAAACTC |  |  |  |  |  |
| Ol13F08 | GTGGACGTTCATGTCCCTTC | 55 | 6 | 1.226 | 0.442 | 0.184 |
|  | CCTGAATCGATTTTCGTCTTG |  |  |  |  |  |
| Ra2E12 | TGTCAGTGTGTCCACTTCGC | 55 | 2 | 1.127 | 0.227 | 0.113 |
|  | AAGAGAAACCCAATAAAGTAGAACC | |  |  |  |  |
| Ra2F11 | TGAAACTAGGGTTTCCAGCC | 55 | 2 | 1.543 | 0.537 | 0.352 |
|  | CTTCACCATGGTTTTGTCCC |  |  |  |  |  |
| Na10D03 | ATGATTTGCCTTGAAATGCC | 55 | 2 | 1.576 | 0.552 | 0.366 |
|  | GATGAAACAATAACCTGAGACACAC | |  |  |  |  |
| Na10D07 | CTACTTTGATGGACACTTGCC | 57 | 3 | 2.664 | 1.036 | 0.625 |
|  | TCTGAAGTTGATTAGTCGGTCC |  |  |  |  |  |
| Na10D09 | AAGAACGTCAAGATCCTCTGC | 55 | 3 | 1.184 | 0.318 | 0.155 |
|  | ACCACCACGGTAGTAGAGCG |  |  |  |  |  |
| Na10H03 | GAGCTGGCTCATTCAACTCC | 55 | 2 | 1.164 | 0.269 | 0.141 |
|  | CACAATTTCTCAGACAAAACGG | |  |  |  |  |
| Na12B09 | ACGGAAGATCAAACAGCTCC | 55 | 2 | 1.025 | 0.067 | 0.024 |
|  | TGAGCGACCCATTCTTTAGG |  |  |  |  |  |
| Na12D03 | GGTAAGCCAAAAACCCTTCC | 60 | 2 | 1.989 | 0.691 | 0.497 |
|  | GAAACCGGTAACAAAGTCGG |  |  |  |  |  |
| Na14E08 | TTACTATCCCCTCTCCGCAC | 55 | 3 | 1.676 | 0.679 | 0.403 |
|  | GCGGATTATGATGACGCAG |  |  |  |  |  |
| Na14G10 | ACGAAGTGGGTTAGTAGGCG | 55 | 4 | 2.078 | 0.794 | 0.519 |
|  | GAAGCCTTTCTCCACCATTG |  |  |  |  |  |
| Na14H11 | GGATGTTTTCACAGACCCTG | 55 | 3 | 2.122 | 0.809 | 0.529 |
|  | CTTTGCAGGTATGAACACGC |  |  |  |  |  |
| Na14H12 | CACATTGGCACGTATCCATC | 55 | 3 | 1.052 | 0.133 | 0.049 |
|  | GGCTGATCGAACACAAATAAG |  |  |  |  |  |
| Ni2F11 | AAAGGGTTTCAATTTCACGC | 55 | 3 | 2.234 | 0.939 | 0.552 |
|  | GGGAAACATACTCACCACGC |  |  |  |  |  |
| BoPLD2 | ACAACCAAACCTGAACACCC | 55 | 2 | 1.401 | 0.461 | 0.286 |
|  | ATCTCAGAGTCCCTTGCACC |  |  |  |  |  |
| BoMT1 | TACGCAGGAAGACTGGTGG | 55 | 2 | 1.019 | 0.054 | 0.019 |
|  | GCAGGAAAATTCAAGGTTAAGAG | |  |  |  |  |
| O112G04b | CGAACATCTTAGGCCGAATC | 55 | 3 | 1.497 | 0.607 | 0.332 |
|  | GGTTAACCTGCGGGATATTG |  |  |  |  |  |
| BoGMS1042 | ATAGTGAATAATGGAAGGCTG | 57 | 2 | 1.466 | 0.498 | 0.318 |
|  | GAGAGAGGAGAGAACAGAGGA |  |  |  |  |  |
| BoCAL | GTTAAGTGTGGCGTTAGAGG | 55 | 2 | 1.515 | 0.523 | 0.340 |
|  | CCTTGGTACATGCCACTGAA |  |  |  |  |  |
| BoDCTD1 | AGAAAGCAGACGGGAATGG | 55 | 3 | 1.336 | 0.473 | 0.252 |
|  | TGGTTAAAGCGAAAGTGTGC |  |  |  |  |  |
| BoKAH45TR | ATTATGACGCCTGGTTTTA | 55 | 2 | 1.921 | 0.673 | 0.479 |
|  | ATTGGTTAGAAGTTATGGGAAC | |  |  |  |  |
| BoIAB94TF | CCAAAGATTCAGAGGAAATGG | 55 | 2 | 1.052 | 0.118 | 0.049 |
|  | GCGTCAAAAACGGTGTCG |  |  |  |  |  |
| SSRB05 | AACGCATCCATCCTCACTTC | 55 | 2 | 1.091 | 0.179 | 0.083 |
|  | AAACCAGCTCGTTCGGTTC |  |  |  |  |  |
| NA8 | ACTGAGAGCAACAACAACAAC | 57 | 2 | 1.947 | 0.680 | 0.486 |
|  | GTAGAGACGGAACCCTGA |  |  |  |  |  |
| Mean |  |  | 2.581 | 1.599 | 0.517 | 0.316 |
| STDEV |  |  | 0.823 | 0.515 | 0.289 | 0.194 |
