# Supplementary material for: The genetic diversity and relationships of cauliflower (Brassica oleracea var. botrytis) inbred lines assessed by using SSR markers
Source: PLoS One. 2018 Dec 6;13(12):e0208551. doi: 10.1371/journal.pone.0208551 (PMC6283626; doi:10.1371/journal.pone.0208551)
Supplement: S4 Table — (DOCX) [file pone.0208551.s004.docx]

**S4 Table. The LnP(D) (log-likelihood) function value calculated by the STRUCTURE program for determining a suitable K value.**

| K | LnP(D) | Var [LnP(D)] | α1 |
| --- | --- | --- | --- |
| 2 | -6380.60 | 172.9 | 0.404 |
| 2 | -6379.60 | 171.5 | 0.406 |
| 2 | -6380.80 | 173.1 | 0.402 |
| 2 | -6383.10 | 177.3 | 0.398 |
| 2 | -6381.00 | 173.6 | 0.402 |
| 2 | -6382.20 | 176.2 | 0.399 |
| 2 | -6380.90 | 173.8 | 0.401 |
| 2 | -6382.10 | 175.9 | 0.400 |
| 2 | -6381.90 | 175.2 | 0.405 |
| 2 | -6381.70 | 175.0 | 0.399 |
| 3 | -6096.40 | 261.3 | 0.176 |
| 3 | -6106.20 | 249.1 | 0.144 |
| 3 | -6096.40 | 257.1 | 0.158 |
| 3 | -6098.00 | 260.8 | 0.161 |
| 3 | -6096.00 | 261.3 | 0.181 |
| 3 | -6097.30 | 259.6 | 0.162 |
| 3 | -6111.40 | 258.3 | 0.145 |
| 3 | -6096.40 | 257.3 | 0.158 |
| 3 | -6095.60 | 257.0 | 0.160 |
| 3 | -6098.20 | 265.0 | 0.181 |
| 4 | -5833.40 | 363.0 | 0.112 |
| 4 | -5831.70 | 360.5 | 0.113 |
| 4 | -5832.10 | 359.4 | 0.112 |
| 4 | -5835.20 | 365.6 | 0.111 |
| 4 | -5830.00 | 354.8 | 0.112 |
| 4 | -5829.80 | 356.0 | 0.112 |
| 4 | -5834.00 | 363.4 | 0.112 |
| 4 | -5833.50 | 363.3 | 0.112 |
| 4 | -5830.90 | 356.8 | 0.111 |
| 4 | -5833.40 | 362.3 | 0.113 |
| 5 | -5722.00 | 406.4 | 0.060 |
| 5 | -5689.10 | 396.9 | 0.061 |
| 5 | -5682.60 | 384.5 | 0.061 |
| 5 | -5688.20 | 394.1 | 0.061 |
| 5 | -5723.00 | 408.5 | 0.062 |
| 5 | -5681.10 | 381.2 | 0.061 |
| 5 | -5687.70 | 393.6 | 0.061 |
| 5 | -5721.50 | 444.6 | 0.078 |
| 5 | -5729.10 | 419.9 | 0.063 |
| 5 | -5815.10 | 615.8 | 0.062 |
| 6 | -5604.40 | 471.0 | 0.046 |
| 6 | -5586.80 | 485.7 | 0.049 |
| 6 | -5581.50 | 479.9 | 0.050 |
| 6 | -5583.70 | 480.4 | 0.050 |
| 6 | -5606.60 | 450.5 | 0.046 |
| 6 | -5604.40 | 473.2 | 0.047 |
| 6 | -5579.60 | 473.0 | 0.050 |
| 6 | -5586.80 | 488.1 | 0.050 |
| 6 | -5584.60 | 482.5 | 0.050 |
| 6 | -5582.80 | 480.4 | 0.049 |
| 7 | -5495.80 | 522.0 | 0.041 |
| 7 | -5521.80 | 570.6 | 0.042 |
| 7 | -5516.80 | 563.2 | 0.042 |
| 7 | -5520.40 | 569.4 | 0.042 |
| 7 | -5526.60 | 581.1 | 0.042 |
| 7 | -5497.40 | 526.7 | 0.041 |
| 7 | -5495.00 | 521.8 | 0.041 |
| 7 | -5496.00 | 521.9 | 0.041 |
| 7 | -5498.30 | 526.2 | 0.041 |
| 7 | -5516.70 | 561.4 | 0.042 |
| 8 | -5433.20 | 595.1 | 0.037 |
| 8 | -5440.10 | 609.4 | 0.037 |
| 8 | -5433.60 | 593.8 | 0.037 |
| 8 | -5436.70 | 601.9 | 0.037 |
| 8 | -5446.30 | 618.5 | 0.037 |
| 8 | -5505.80 | 659.7 | 0.040 |
| 8 | -5436.40 | 599.6 | 0.037 |
| 8 | -5446.60 | 619.5 | 0.037 |
| 8 | -5442.60 | 609.3 | 0.037 |
| 8 | -5510.00 | 660.5 | 0.039 |
| 9 | -5407.10 | 668.0 | 0.036 |
| 9 | -5424.80 | 678.6 | 0.036 |
| 9 | -5409.90 | 654.8 | 0.036 |
| 9 | -5419.90 | 676.4 | 0.036 |
| 9 | -5411.20 | 675.7 | 0.036 |
| 9 | -5420.10 | 676.2 | 0.036 |
| 9 | -5435.50 | 701.3 | 0.036 |
| 9 | -5538.90 | 828.8 | 0.035 |
| 9 | -5465.40 | 657.5 | 0.035 |
| 9 | -5533.70 | 841.0 | 0.035 |
| 10 | -5402.00 | 761.3 | 0.035 |
| 10 | -5442.60 | 732.7 | 0.034 |
| 10 | -5561.70 | 973.7 | 0.034 |
| 10 | -5466.40 | 790.8 | 0.034 |
| 10 | -5435.30 | 721.6 | 0.034 |
| 10 | -5394.50 | 751.9 | 0.035 |
| 10 | -5558.20 | 861.0 | 0.033 |
| 10 | -5499.20 | 889.0 | 0.034 |
| 10 | -5521.10 | 933.4 | 0.035 |
| 10 | -5466.80 | 657.5 | 0.032 |
